# Fellow-Workers and the Roll of Honour

**Published:** 1915-01-23

**Authors:** 


### ROUND THE WORLD.

[The Editor Invites Early Information and Contributions Marked "Fellow-Workers."]

### Fellow-Workers and the Roll of Honour.

It is said to be more than ten years since an officer of the Indian Medical Service has been killed in action, the late Captain F. Syme, who met his death in Somaliland in 1903, being the last I.M.S. man to have laid down his life for his country in this way. Now the deaths are recorded of two capable officers of this fine service, Major P. P. Atal and Captain K. I. Singh, and it will be indeed a fortunate thing if its keen officers do not suffer in the further hard work which will undoubtedly fall to the share of the Indian Expeditionary Force before the war is over.

Major Pundit Piaraylal Atal, I.M.S., was a "Bart.'s" man, who qualified in 1898, when he was twenty-six years of age, by taking the L.R.C.P. Lond., M.R.C.S. Eng. A keen worker, he devoted much time to the improvement of his clinical experience before settling down to his life's career, and held the appointments of house physician and junior house surgeon in the Clayton Hospital at Wakefield. Entering the I.M.S. in 1899, he soon saw active service, going through the China war in the following year, and subsequently receiving a medal for his services at that time. He attained his majority three years ago, and was attached to the 129th Beluchis when the war broke out.

Captain Kanwar Indarjit Singh, I.M.S., was a much younger man than the last-named, being only thirty-one years of age at the time of his death. Indeed, he only qualified three years ago, after having fulfilled the medical curriculum at Cambridge and King's College Hospital. Having become an M.B., B.C. Cantab., he aimed at the higher distinction of M.R.C.P. Lond., and was successful in gaining his object after only about a year's further study. Captain Singh was already at that time a lieutenant in the I.M.S., which service he entered immediately after qualification; he was promoted to captain last spring, and had been for some little time medical officer to the 57th Rifles.

Dr. James Collier, who has taken on responsible work in connection with the wounded soldiers, both as one of the physicians to the King George Hospital and to the Special Hospital for Wounded Officers suffering from Nervous Shock, has been for many years a keen investigator of neurological problems. A student of St. Mary's, where he won many distinctions, including the gold medal in anatomy at the Intermediate M.B. Lond., and the gold medal in forensic medicine at the M.B. Lond., Dr. Collier subsequently joined the resident staff of the National Hospital for the Paralysed and Epileptic in Queen Square, an institution with which he has since maintained his connection, working through various junior posts to his present position of physician in charge of out-patients. He is also physician and lecturer on medicine at St. George's Hospital and physician to the Royal Eye Hospital.

The staff of the King George Hospital also includes Dr. Fleming Mant Sandwith, whose first Chadwick lecture on "War and Disease" appears on page 375. An M.D. Durham, F.R.C.P. Lond., and an authority on tropical diseases, Dr. Sandwith is senior physician to the Albert Dock Hospital, lecturer at the London School for Tropical Medicine and at St. Thomas's Hospital, as well as examiner in his special subject at London University, and Chairman of the County of London Branch of the British Red Cross Society. In early days he saw much fighting in the Near East, having served as ambulance surgeon during the Turko-Servian war of 1876, and the subsequent Russo-Turkish campaign when he was present at the famous Shipka Pass action. Proceeding to Egypt later on he assisted in combating the cholera epidemic in 1883, and became Vice-Director of the Sanitary Department of the Egyptian Government. After some years he was appointed Professor of Medicine in the Cairo School of Medicine, and again saw active service at the time of the South African war, when he was senior physician to the Imperial Yeomanry Hospital at Pretoria.

The late Captain Brian Colden Antill Pockley, who was killed in action during the successful attack of the Australian Expeditionary Force on Herbertshohe, in German New Guinea, was a popular student of Sydney University, where he qualified M.B., Ch.M. only a few months ago, his father being Dr. F. Antill Pockley, a well-known Sydney practitioner, who was President of the Australasian Medical Congress held there in 1911. Joining the Expeditionary Force as surgeon from the Australian Naval Reserve, this unfortunate young officer has lost his life at the early age of twenty-four years through his splendidly unselfish action in protecting with his own Red Cross coat a sailor who had been sent off in charge of a wounded comrade. Working thereafter in his shirt sleeves, the late Captain Pockley was apparently mistaken for a combatant officer and shot down.

Lieutenant David Wylie Rintoul, R.A.M.C., also killed in action, received his medical training in St. Andrews University, where he graduated M.B., Ch.B. two years ago. After qualification he was successful in gaining a resident appointment at the Bristol General Hospital, where he was successively house surgeon and house physician, and performed his duties with such thoroughness that on giving up work there to enter the R.A.M.C. he was specially thanked by the committee for his services. He had been attached to the Eastern Command before being ordered to the Continent.

The staff of the Special Hospital for Wounded Officers suffering from Nervous Shock includes several prominent advocates of the treatment by hypnotism and suggestion. Of these, Dr. John Milne Bramwell, M.B., C.M. Edin., has done as much as anyone in this country to develop the scientific study of hypnotism, his works on that subject being widely read. Originally in private practice at Goole, in Yorkshire, Dr. Bramwell's hypnotic researches attracted so much attention that he found himself compelled to devote the greater part of his time to this special branch, and eventually gave up general work and settled in London. His researches include important experiments on hypnotic anæsthesia and the sub-conscious estimation of time; while his leading work is "Hypnotism, its History, Practice, and Theory," originally published in 1903.

